# The immunomodulatory effect of cathelicidin-B1 on chicken macrophages

**DOI:** 10.1186/s13567-020-00849-y

**Published:** 2020-09-24

**Authors:** Lianci Peng, Maaike R. Scheenstra, Roel M. van Harten, Henk P. Haagsman, Edwin J. A. Veldhuizen

**Affiliations:** Department of Biomolecular Health Sciences, Division of Infectious Diseases & Immunology, Section of Molecular Host Defence, Faculty of Veterinary Medicine, Utrecht University, Yalelaan 1, 3584CL Utrecht, The Netherlands

**Keywords:** chicken, innate immunity, macrophages, antimicrobial peptides, cathelicidins, *E. coli*

## Abstract

Cathelicidins (CATHs) play an important role in the innate immune response against microbial infections. Among the four chicken cathelicidins, CATH-B1 is studied the least. In this study, the effect of CATH-B1 on the macrophage response towards avian pathogenic *E. coli* (APEC) and bacterial ligands was investigated. Our results show that APEC induced CATH-B1 gene expression in both a chicken macrophage cell line (HD11 cells) and primary macrophages, while expression of the other three CATHs was virtually unaffected. While the antimicrobial activity of CATH-B1 is very low under cell culture conditions, it enhanced bacterial phagocytosis by macrophages. Interestingly, CATH-B1 downregulated APEC-induced gene expression of pro-inflammatory cytokines (IFN-β, IL-1β, IL-6 and IL-8) in primary macrophages. In addition, CATH-B1 pre-incubated macrophages showed a significantly higher gene expression of IL-10 after APEC challenge, indicating an overall anti-inflammatory profile for CATH-B1. Using isothermal titration calorimetry (ITC), CATH-B1 was shown to bind LPS. This suggests that CATH-B1 reduces toll like receptor (TLR) 4 dependent activation by APEC which may partly explain the decreased production of pro-inflammatory cytokines by macrophages. On the contrary, direct binding of CATH-B1 to ODN-2006 enhanced the TLR21 dependent activation of macrophages as measured by nitric oxide production. In conclusion, our results show for the first time that CATH-B1 has several immunomodulatory activities and thereby could be an important factor in the chicken immune response.

## Introduction

Cathelicidins are host defense peptides (HDPs) with antimicrobial activity and immunomodulatory functions. They are produced as inactive precursors (prepropeptides), stored in granules, and upon cell activation released as mature peptides by proteolytic cleavage [1]. Cathelicidins have been found in many different species, including mammals, reptiles, amphibians, fishes and birds [2, 3]. Interestingly, the number of functional genes encoding cathelicidins in different species is highly variable [4]. For example, only a single cathelicidin (LL-37) is present in human, while chicken has four cathelicidins with varying length and structure (CATH-1, -2, -3 and -B1) [5–8].

Of the four chicken cathelicidins, CATH-2 has been studied extensively. CATH-2 has broad antimicrobial activity and strong immunomodulatory effects, such as lipopolysaccharide (LPS) binding, neutralization of the immune response and enhanced DNA-induced activation of toll like receptor (TLR) 21 [9–12]. In addition, in ovo administration of the all D-amino acid enantiomer of CATH-2 (D-CATH-2) at embryonic day 18 resulted in a protective effect against avian pathogenic *E. coli* (APEC) infection up to 7 days after hatch [13]. However, less is known about the activities of CATH-B1, which means that it is challenging to properly compare functionalities and activities [14]. When comparing expression patterns, one clear difference between CATH-1, -2, -3 and CATH-B1 is apparent: using immunostaining and mass spectrometry CATH-1, -2, -3 were detected in heterophils [15], while CATH-B1 was shown to be produced by epithelial cells in the bursa of Fabricius, although an extended description of CATH-B1 protein expression among cells/tissues was not described [6].

APEC is an important pathogen that causes severe respiratory diseases in chicken, leading to huge economic losses in poultry farming. APEC infection starts in the trachea and damages the respiratory mucosa. Subsequently, it crosses the epithelial layer and enters the blood stream spreading to other tissues [16]. APEC can be phagocytosed by macrophages both in the lungs and in the blood stream, which leads to (partial) killing of the pathogen, but also induces an immune response that attracts other immune cells such as heterophils to infected sites.

Compared to mammalian lungs, the healthy chicken lung has a relatively low number of macrophages, but a large increase in number of macrophages occurs in the lung and air sacs after APEC infection [17–19]. This suggests that macrophages play an important role against microbial infection in the chicken lung. Interestingly, bacterial metabolites such as butyrate induced the gene expression of CATH-B1 in chicken macrophages including HD11 cells and primary monocytes, whereas gene expression of the other three cathelicidins was very low in macrophages compared to heterophils [20]. This is an indication that CATH-B1 might play an important role in macrophages upon interaction with *E. coli*.

Therefore, in this study, our main aim was to investigate the effect of CATH-B1 on chicken macrophages and their response towards APEC and TLR agonists. We found that gene expression of CATH-B1 was induced by APEC in both HD11 cells and blood monocyte-derived macrophages. CATH-B1 enhanced phagocytosis of APEC by macrophages. Furthermore, CATH-B1 inhibited APEC- and LPS- induced immune response but enhanced DNA-induced nitric oxide (NO) in macrophages. Our study provides additional insights in the functions of CATH-B1, that are clearly different from those of the other chicken cathelicidins.

## Methods and materials

### Peptides

All peptides were synthesized by China Peptides (Shanghai, China) using Fmoc-chemistry and purified by reverse phase high-performance liquid chromatography to a purity of > 95%.

### Bacterial strains

The APEC strain is a clinical isolate from chicken [13]. Heat-killed bacteria were prepared by heating the bacterial suspension at 75  °C for 15 min. Viability was checked by plating out on Tryptic Soy Agar (TSA) (Oxoid, Basingstoke, UK) plates.

### Antimicrobial activity assay

APEC was cultured in Tryptic Soy Broth (TSB) (Oxoid, Basingstoke, UK) at 37 °C and grown to mid-logarithmic growth phase before testing. Bacterial suspensions were pelleted by centrifugation, resuspended in TSB or Gibco cell culture medium (RPMI1640 or DMEM + glutamax (Thermo Fischer Scientific, Waltham, MA, USA)) with 10% FCS (Corning, Glendale, USA) and diluted to 2.0 × 10^6^ CFU/mL. Twenty-five μL of cathelicidins (0–80 μM) were mixed with an equal volume of bacterial suspension and incubated for 3 h at 37 °C. After incubation, dilution series of bacteria were plated out on TSA plates and incubated at 37  °C for 24 h to quantify viable bacteria.

### Cell culture

Monocyte-derived macrophages were obtained as described before [21]. In short, peripheral blood mononuclear cells (PBMCs) were isolated from blood of 76-week-old healthy chickens using Ficoll density gradient centrifugation and were frozen in liquid nitrogen until use. PBMCs (1 × 10^7^ cells) were seeded in a 24-well plate and incubated at 41 °C (5% CO_2_). After overnight culture, all non-attached cells were removed and attached cells (monocytes) were maintained in RPMI1640 + glutamax medium with 10% FCS and 1% P/S (100 U penicillin/mL; 100 µg streptomycin/mL (Thermo Fischer Scientific, Waltham, MA, USA) supplemented with recombinant chicken GM-CSF produced in COS-7 cells for another 2 days at 41 °C (5% CO_2_) [22]. These monocyte-derived macrophages were used for further analysis.

The chicken macrophage cell line HD11 was maintained in RPMI1640-glutamax supplemented with 10% FCS and 1% P/S at 41 °C (5% CO_2_). HD11 cells were seeded in a 24-well plate (2.5 × 10^5^ cells/well) or 96-well plate (0.5 × 10^5^ cells/well) and cultured overnight to adhere before further analysis.

Mouse macrophages (RAW 264.7 cells) were maintained in DMEM-glutamax supplemented with 10% FCS at 37 °C (5% CO_2_). RAW cells were seeded in a 96-well plate (0.5 × 10^5^ cells/well) and cultured overnight to adhere before further analysis.

### Cell viability

Cell viability was determined using the WST-1 assay following the manufacturer’s protocol. Briefly, primary macrophages were incubated with cathelicidins for 3 h at 41 °C (5% CO_2_). Subsequently, cells were washed and further incubated for 3 h at 41 °C. Cell culture medium was removed and replaced with fresh culture medium containing 10% WST-1 reagent (Roche, Mannheim, Germany). After 20 min incubation, absorbance was measured at 450 nm with a FLUOstar Omega microplate reader and was corrected for absorbance at 630 nm.

### APEC infection in chicken macrophages

Primary macrophages and HD11 cells were cultured as described above. Aliquots of 0.5 mL of bacterial suspensions (1  ×  10^6^ CFU/mL) were added to each well in the presence or absence of 5 µM CATH-B1 or CATH-2, with three replicate wells for a 24-well plate and incubated for 3 h at 41 °C (5% CO_2_). In phagocytosis studies, bacteria were removed at 3 h post infection and cells were washed three times with RPMI1640-glutamax medium with 10% FCS. Then, RPMI 1640-glutamax containing 500 μg/mL gentamicin (Sigma-Aldrich) was added to cells in order to kill all extracellular, non-phagocytosed bacteria and plates were put back at 41 °C for 1 h. Infected cells were washed three times with RPMI1640-glutamax and lysed by 0.5 mL 0.5% Triton X-100. After lysis, dilution series of cells were plated on TSA plates and incubated at 37  °C for 24 h to quantify viable bacteria.

In pre-incubation studies, CATH-B1 was added to primary macrophages for 3 h, washed away with cell culture medium after which APEC were added for 3 h. In post-incubation studies, APEC were added to primary macrophages for 3 h, washed away and infected cells were treated with CATH-B1 with gentamicin for 3 h. After that, cells were treated with TRIzol (Thermo Fisher Scientific, Waltham, MA, USA) for RNA isolation.

### LPS and ODN-2006 stimulation

Primary macrophages and RAW cells were cultured as described above. UltraPure LPS *E. coli* O111:B4 (100 ng/mL) (InvivoGen, San Diego, CA, USA), was diluted in RPMI1640-glutamax medium with 10% FCS, and added to cells in the presence or absence of 5 μM CATH-B1 or CATH-2 for 4 h. Afterwards, primary macrophages were washed and treated with TRIzol for RNA isolation.

HD11 cells were prepared in a 96-well plate as described above. ODN-2006 (5 nM) (InvivoGen, Toulouse, France) was added to HD11 cells in the presence or absence of different concentrations (0–10 μM) of CATH-B1 or CATH-2 for 20 h. After this incubation, cell supernatants were collected to measure NO production (see below).

### Quantitative real-time PCR (qPCR)

**Figure 1:**
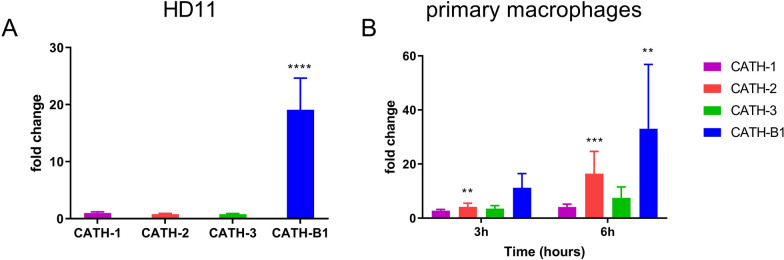
**APEC-induced gene expression of chicken cathelicidins (CATHs).** Cells were infected with APEC for 3 h (primary macrophages and HD11 cells) or 6 h (primary macrophages). After that, RNA was isolated to determine gene expression of CATHs by qPCR analysis in **A** HD11 cells and **B** primary macrophages. Data are represented as mean  ±  SEM of three independent experiments of triplicate samples per experiment. **P ≤ 0.01;***P ≤ 0.005; ****P ≤ 0.001.

Primary macrophages were treated with APEC and LPS as described above. After incubation, total RNA was extracted by TRIzol reagent according to the manufacturer’s instructions. RNA (500 ng) was reverse transcribed by the iScript cDNA synthesis kit (Bio-Rad, Veenendaal, the Netherlands) according to the manufacturer’s instructions. Primers and probes were designed and produced by Eurogentec (Seraing, Belgium) (Table 1). qPCR was performed in clear thin-walled 96 well plates (Biorad, Hercules, CA, USA) using adhesive seals (Biorad ‘B’ seals) on a CFX Connect qPCR with CFX Manager 3.0 (Bio-Rad). All reactions were performed in Biorad IQ supermix (or IQ Sybrgreen supermix for CATH-2) with 300 nM forward and reverse primer and 100 nM probe and 25 ng cDNA as template. Cycling conditions were similar for all PCR reactions: 3 min at 95 °C; 40 cycles: 10 s at 95 °C, 30 s at 60 °C and 30 s at 72 °C, resulting in cycling efficiencies between 90% and 110%. Relative gene expression levels were normalized against the expression levels of the reference genes GAPDH and 28S. Under the conditions used in this study these two reference genes, and also the reference genes chicken ribosomal protein S17 and chicken RAS related protein RAB14, did not change compared to each other. Results are shown as ‘fold change’: expression compared to either unstimulated cells (Figure 1) or as relative expression compared to a specific subset of stimulated cells (Figures 6, 7 and 8) due to undetectable levels of cytokine gene expression in control non-stimulated cells, hampering calculation of relative expression levels compared to unstimulated controls.

**Table 1:** Primer and probe sequences for qPCR

Gene	5′ → 3′sequence	Accession number
GAPDH	F: GTCAACCATGTAGTTCAGATCGATGA	NM_204305.1
	R: GCCGTCCTCTCTGGCAAAG	
	P: AGTGGTGGCCATCAATGATCCC	
28S	F: GGCGAAGCCAGAGGAAACT	X59733
	R: GACGACCGATTTGCACGTC	
	P: AGGACCGCTACGGACCTCCACCA	
IFN-β	F: CCTCCAACACCTCTTCAACACG	KF741874.1
	R: TGGCGTGTGCGGTCAAT	
	P: AGCAGCCCACACACTCCAAAACACT	
IL-1β	F: GCTCTACTAGTCGTGTGTGATGAG	NM_204524.1
	R: TGTCGATGTCCCGCATGA	
	P: CCACACTGCAGCTGGAGGAAGCC	
IL-6	F: GTCGAGTCTCTGTGCTAC	NM_204628.1
	R: GTCTGGGATGACCACTTC	
	P: ACGATCCGGCAGATGGTGA	
IL-8	F: GCCCTCCTCCTGGTTTCA	NM_205498.1
	R: CGCAGCTCATTCCCCATCT	
	P: TGCTCTGTCGCAAGGTAGGACGCTG	
IL-10	F: CATGCTGCTGGGCCTGAA	NM_001004414.2
	R: CGTCTCCTTGATCTGCTTGATG	
	P: CGACGATGCGGCGCTGTCA	
CATH-1	F: GCTGACCCTGTCCGCGTCA	NM_001001605.3
	R: GAGGTTGTATCCTGCAATCAC	
	P: CCTGATGACCAGCGGC	
CATH-2	F: CAAGGAGAATGGGGTCATCAG	NM_001024830.2
	R: CGTGGCCCCATTTATTCATTCA	
CATH-3	F: CCATGGCTGACCCTGTCC	NM_001311177.1
	R: TGATGGCTTTGTAGAGGTTGATG	
	P: CGCAGCCACCGTGTTG	
CATH-B1	F: TGTTCCATAGATCAGCAG	NM_001271172.1
	R: ATTCAACCACTCCCAGATG	
	P: TCCACCAGTTGCGGAT

### Griess assay

HD11 cells were stimulated with ODN-2006 as described above. Supernatant was collected to measure NO production. Briefly, 30 μL of sample were added to the well in a 96-well flat bottom plate. An equal volume of 1% sulfanilamide (Merck, Darmstadt, Germany) was added in each well, followed by 30 μL 0.1% N-(1-naphthyl) ethylenediamine dihydrochloride (VWR) at room temperature for 5 min. The nitrite concentration was determined by measuring optical density at 550 nm. Sodium nitrite (Merck, Darmstadt, Germany,) was used as a standard to accurately determine the nitrite concentration in the cell supernatant.

### Isothermal titration calorimetry

Interaction between CATH-B1 and *E. coli* LPS O111:B4 or ODN-2006 was tested using isothermal titration calorimetry (ITC). All ITC experiments were performed on a Low Volume NANO ITC (TA instruments - Waters LLC, New Castle, USA). LPS was diluted in PBS to 0.5 mg/mL in PBS/H_2_O, 3:1 v/v (75% PBS), rigorously vortexed for 5 min and added to the cell chamber (167 µL). ODN-2006 was diluted to 25 nM in 75% PBS. The syringe was filled with a 50 µL solution of 200 µM CATH-B1 in 75% PBS. Titrations were incremental with 2 µL injections (for LPS) or 1 µL injections (for ODN-2006) at 300 s intervals. Experiments were performed at 37 °C and data were analyzed with the Nano Analyze software (TA instruments - Waters LLC).

### ELISA

RAW 264.7 cells were prepared in a 96-well plate as described above. RAW cells were stimulated with *E. coli* LPS (100 ng/mL) in the presence or absence of 5 µM CATH-B1 or CATH-2 for 24 h. Cell supernatants were collected to measure cytokine expression. The mouse IL-6 ELISA kit (R&D Systems, Minneapolis, MN) was used to determine the IL-6 concentration of samples. This assay was performed following the manufacturer’s protocol.

### Statistical analysis

Data are represented as mean  ±  SEM of three independent experiments for each group (n  =  3) and were analyzed by a T-test for two groups or by one-way ANOVA with Tukey’s multiple comparisons test for more than two groups. Bio-Rad CFX Manager 3.0 software was used for qPCR data analysis. All the graphs were made using GraphPad Prism^®^ 8.0.

## Results

### APEC induced CATH-B1 gene expression in macrophages

CATH-B1 protein has so far only been detected in the bursa of Fabricius, but CATH-B1 mRNA is found in different tissues including the respiratory tract, gastrointestinal tract and lymphoid organs [23], indicating that a broader expression of CATH-B1 is likely. In our study, APEC significantly induced gene expression of CATH-B1 in both HD11 cells (Figure 1A) and chicken primary macrophages (Figure 1B). At 6 h post-infection, up to a 30-fold increase in CATH-B1 gene expression was detected in primary macrophages compared to non-infected cells (Figure 1B). Gene expression of CATH-1, -2, -3 was not or only mildly (CATH-2) induced by APEC in both cell types (Figures 1A, B).

### The effect of CATH-B1 on phagocytosis in macrophages

**Figure 2:**
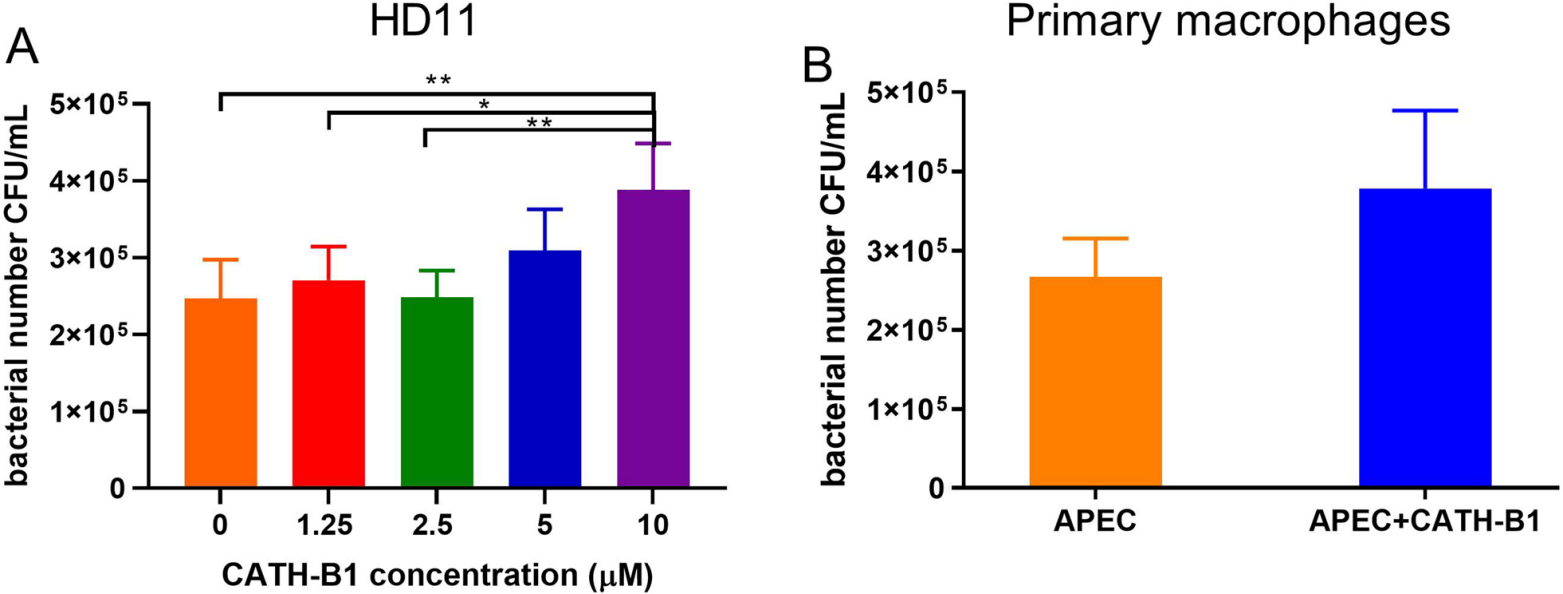
**The effect of CATH-B1 on bacterial phagocytosis in chicken macrophages.** Cells were infected with APEC for 3 h in the presence of 0–10 μM CATH-B1 (HD11 cells) or 5 μM CATH-B1 (primary macrophages), then gentamicin was added to kill extracellular bacteria for 1 h. Finally, cells were lysed to quantify intracellular bacteria in **A** HD11 cells and **B** in primary macrophages. Data are represented as mean  ±  SEM of three independent experiments of triplicate samples per experiment. *P ≤ 0.05; **P ≤ 0.01.

To determine the effect of CATH-B1 on the function of macrophages, the peptide was added to primary macrophages or HD11 cells together with APEC. Bacterial phagocytosis by macrophages was concentration-dependent, significantly enhanced in HD11 cells (Figure 2A) when CATH-B1 was present, and slightly enhanced in primary macrophages (Figure 2B).

### Antibacterial activity of chicken cathelicidins against APEC

**Figure 3:**
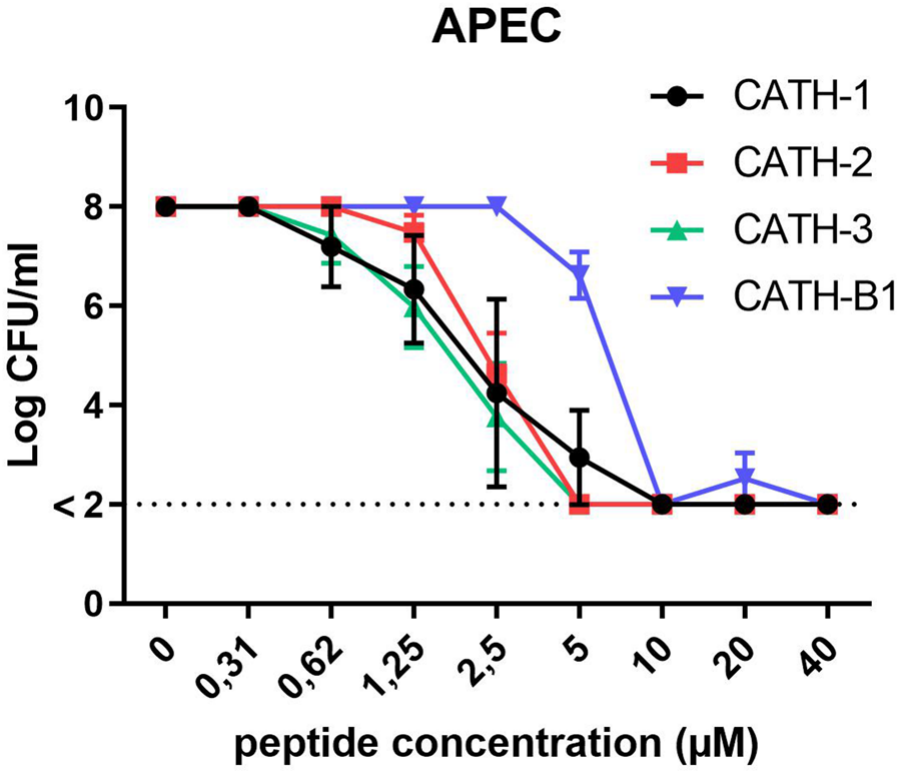
**Antibacterial activity of chicken cathelicidins against APEC.** Bacteria were incubated with different concentrations of cathelicidins for 3 h, serially diluted and spread plated on TSA plates to quantify viable bacteria. Data are represented as mean  ±  SEM of three independent experiments of triplicate samples per experiment.

The antimicrobial activity against APEC in various culture media was tested for the four chicken cathelicidins (CATH-1, -2, -3 and -B1) in order to determine the minimal bactericidal concentration (MBC). This showed that CATH-1, -2, -3 had similar antibacterial activity with MBC values between 5 µM and 10 µM. In contrast with the other three cathelicidins, CATH-B1 showed a weaker anti-APEC activity at 5 μM but killed all bacteria at 10 μM (Figure 3). In cell culture conditions (DMEM + glutamax with 10% FCS), CATH-2 still showed strong antibacterial activity, whereas the antibacterial activity of CATH-1 and CATH-3 was strongly reduced. CATH-B1 completely lost its antibacterial activity showing no growth inhibition of APEC at the highest concentration tested (40 µM) (Additional file 1).

### Cytotoxicity of CATH-B1

**Figure 4:**
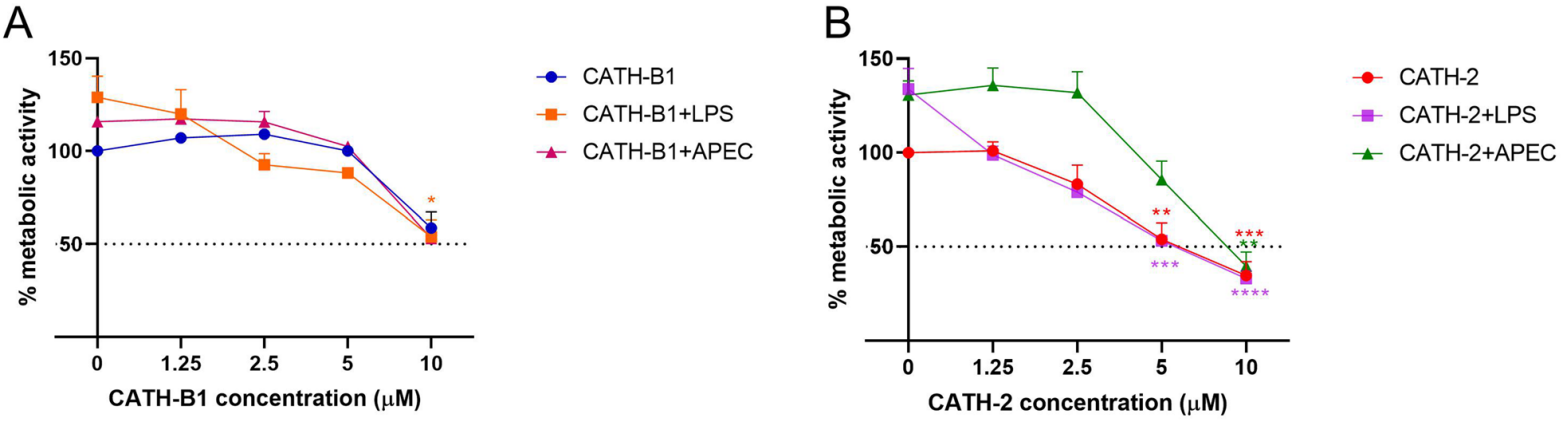
**Cytotoxicity of chicken CATH-B1 and CATH-2.** Primary macrophages were incubated with different concentrations (0–10 μM) of CATH-B1 (**A**) and CATH-2 (**B**) for 3 h in the presence or absence of 100 ng/mL LPS or 1  ×  10^6^ CFU/mL APEC. Cathelicidins were washed away and cells were maintained in new medium for another 3 h. Metabolic activity was tested using WST-1 reagent. Data are represented as mean  ±  SEM of three independent experiments of triplicate samples per experiment. *P ≤ 0.05; **P ≤ 0.01;***P ≤ 0.005; ****P ≤ 0.001.

To determine the toxic effect of CATH-B1 on host cells, the WST-1 assay was used to measure metabolic activity of primary macrophages. CATH-2 was used as a control in this study. It induced cell damage at 5 µM, at which a 40% reduction in metabolic activity was detected. CATH-B1 was less toxic than CATH-2 but reduced metabolic activity at concentrations of 10 µM or higher (Figure 4). LPS and APEC actually increased metabolic activity when present, but did not have a significant effect on metabolic activity after co-incubation with cathelicidins.

### The effect of CATH-B1 on APEC-induced cytokine expression in macrophages

**Figure 5:**
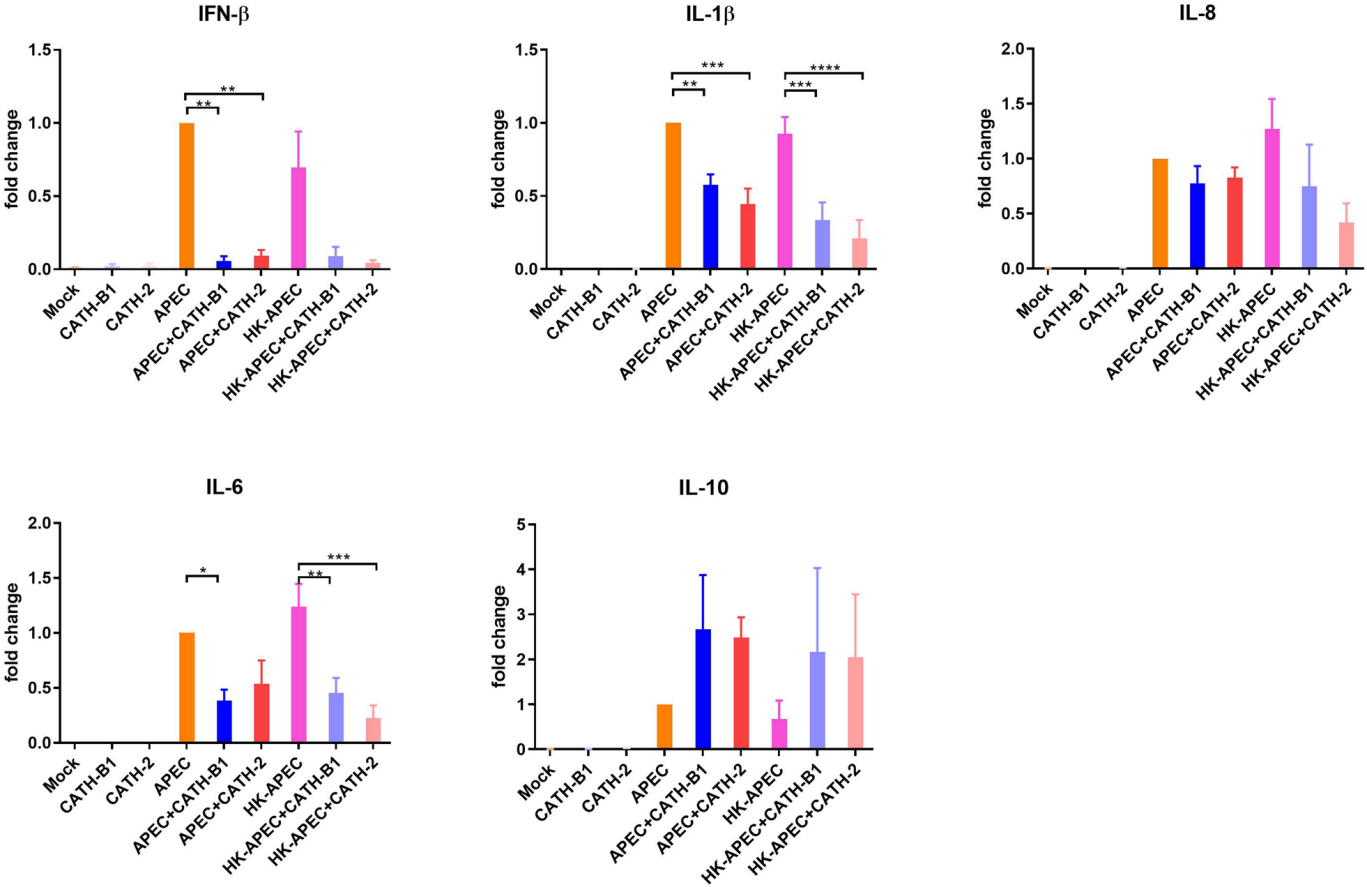
**The effect of CATH-B1 on APEC-induced cytokine expression in primary macrophages.** Gene expression of cytokines in primary macrophages was determined by qPCR at 3 h post infection in the presence or absence of 5 µM CATH-2 and CATH-B1. Data are represented as mean  ±  SEM of three independent experiments of triplicate samples per experiment. *P ≤ 0.05; **P ≤ 0.01; ***P ≤ 0.005; ****P ≤ 0.001.

Activation of macrophages resulting in the release of cytokines is a key immune response against pathogens. However, overexpression of inflammatory cytokines can cause apoptosis of cells leading to tissue damage. Therefore, it is important to have a balanced response of the immune system with respect to release of these cytokines. To investigate whether CATH-B1 regulates APEC-induced activation of macrophages, APEC-induced cytokine expression in the presence or absence of CATH-B1 (and CATH-2 as control) was determined using qPCR. To separate immunomodulatory effects from antibacterial activity of CATH-B1, heat-killed APEC was also used in this experiment. At 3 h post infection, both viable and heat-killed APEC strongly up-regulated gene expression of pro-inflammatory cytokines IL-1β and IL-6, chemokine IL-8 and the anti-inflammatory cytokine IL-10 (Figure 5) compared to control cells that were mock-treated with medium. Both CATH-B1 and CATH-2 downregulated gene expression of these cytokines. Gene expression of IFN-β was also upregulated by APEC and both CATH-B1 and CATH-2 significantly inhibited IFN-β expression (Figure 5). Interestingly, APEC-induced gene expression of IL-10 was increased by CATH-B1 and CATH-2 (Figure 5) but the increase was not significant.

**Figure 6:**
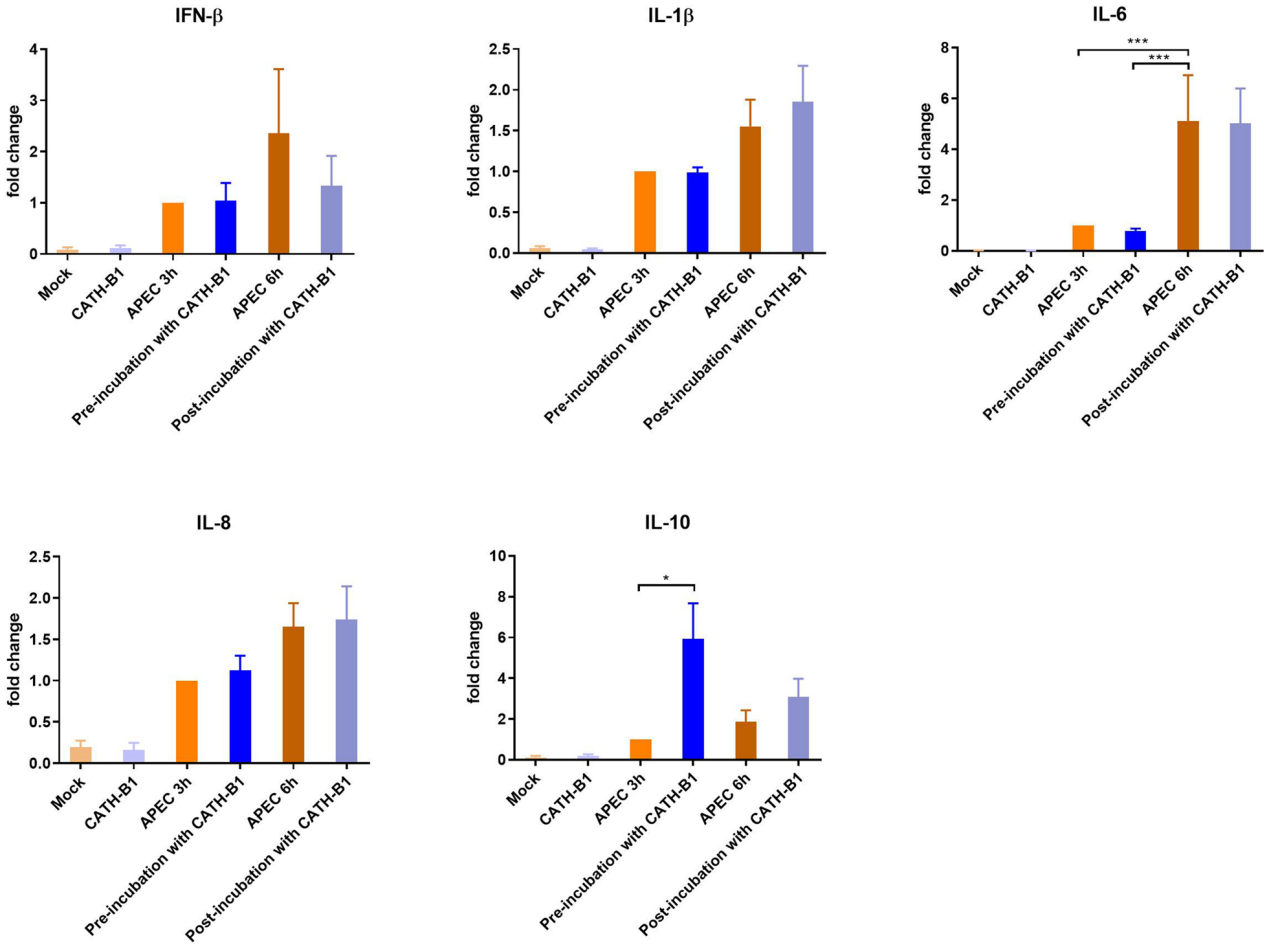
**APEC-induced cytokine expression in primary macrophages upon pre- and post-incubation of CATH-B1.** Primary macrophages were pre-incubated with 5 µM CATH-B1 for 3 h before APEC infection, or post-incubated to 5 µM CATH-B1 for 3 h after APEC infection. Then, gene expression of cytokines was determined by qPCR. Data are represented as mean  ±  SEM of three independent experiments of triplicate samples per experiment. *P ≤ 0.05; ***P ≤ 0.005.

To investigate how CATH-B1 inhibited APEC-induced activation, primary macrophages were pre-incubated with cathelicidins prior to or post APEC infection. APEC-induced gene expression after 3 and 6 h (depending on the setup of the experiment) was similar for IFN-β, IL-1β, IL-8 and IL-10, except for IL-6 gene expression, which was significantly higher after 6 h (Figure 6). The inhibitory effect of CATH-B1 on cytokine gene expression, observed in co-incubation conditions, was lost in pre- and post-incubation conditions. Noticeably, there was one exception, macrophages pre-incubated with CATH-B1 expressed significantly more IL-10 compared to macrophages without CATH-B1. Overall, this indicates an anti-inflammatory effect of CATH-B1 on APEC-infected macrophages.

### The effect of CATH-B1 on LPS-induced cytokines expression in macrophages

**Figure 7:**
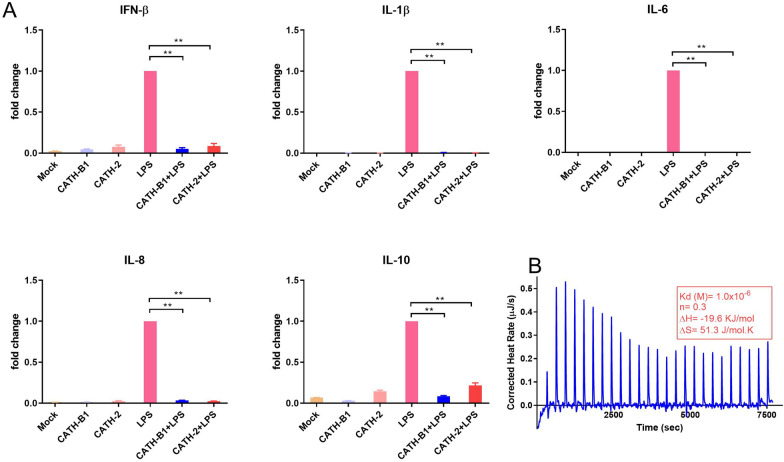
**The effect of CATH-B1 on LPS-induced cytokine expression in primary macrophages.** Primary macrophages were stimulated with LPS (100 ng/mL) for 4 h in the presence or absence of 5 µM CATH-2 or CATH-B1. **A** Gene expression of cytokines. Data are represented as mean  ±  SEM of three independent experiments of triplicate samples per experiment. **P ≤ 0.01. **B** ITC thermogram of CATH-B1 binding to LPS *E. coli* O111:B4. ITC analysis data were calculated as K_d_ = 1.0 × 10^−6^ M, ΔH = − 19.6 kJ/mol and ΔS = 51.3 J/mol.

To further investigate the functional properties of CATH-B1, *E. coli* LPS-induced cytokine gene expression in the presence or absence of cathelicidins was determined. Again, in these experiments CATH-2 was used as a positive control since CATH-2 has been described to neutralize LPS and that CATH-2- LPS binding was essential for this (Van Dijk et al. [30]). LPS-induced gene expression of IFN-β, IL-1β, IL-6, IL-8 and IL-10 was significantly downregulated by both CATH-B1 and CATH-2 (Figure 7A). To investigate whether the inhibitory effect of CATH-B1 is host cell specific, we also tested IL-6 protein production in LPS-stimulated mouse macrophages using ELISA. Also, in mouse macrophages, the cytokine production was inhibited by CATH-B1 (Additional file 2), suggesting that the inhibition is due to the interaction of CATH-B1 and LPS. Finally, ITC analysis was used to determine the direct interaction of CATH-B1 and LPS (Figure 7B). Peptide binding to LPS was detected with an observed dissociation constant of Kd = 1.0 µM in a reaction driven by both enthalpy (ΔH = − 19.6 kJ/mol) and entropy ΔS = 51.3 J/mol). This indicates that CATH-B1 inhibits LPS-induced cytokine expression in macrophages by binding to and thereby neutralizing LPS, similar as has been described for CATH-2.

### The effect of CATH-B1 on DNA-induced NO production in HD11 cells

**Figure 8:**
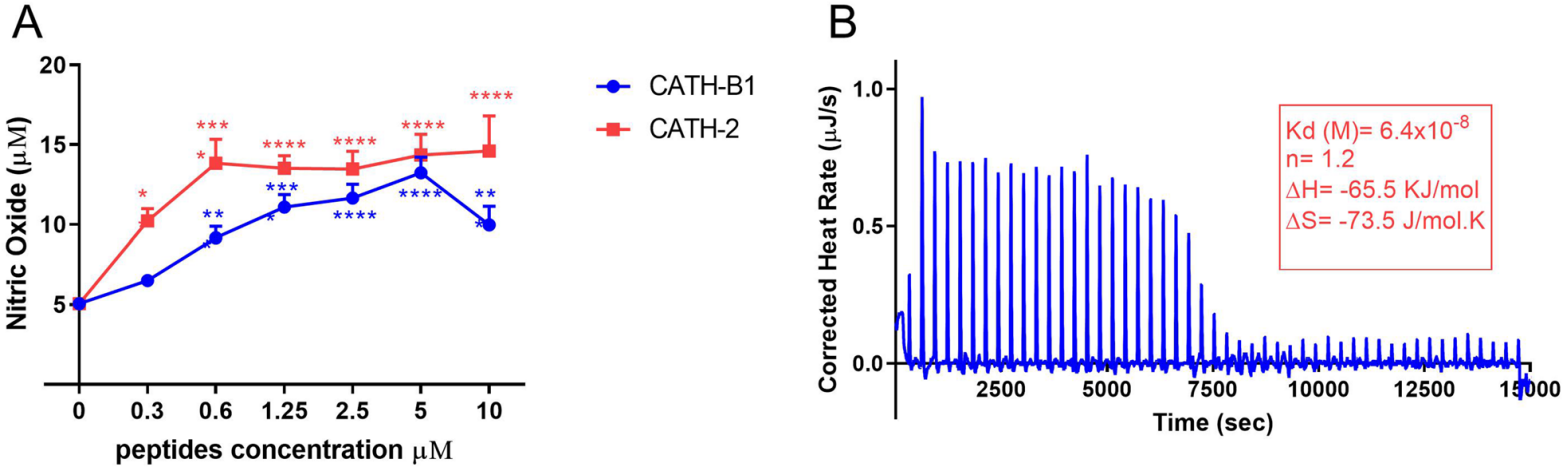
**The effect of CATH-B1 on DNA-induced nitric oxide production in HD11 cells.** HD11 cells were stimulated with 5 nM ODN-2006 for 20 h in the presence or absence of different concentrations of cathelicidins. **A** The amount of NO in the cell supernatant were measured by Griess assay. Data are represented as mean  ±  SEM of three independent experiments of triplicate samples per experiment. **P ≤ 0.01; ***P ≤ 0.005; ****P ≤ 0.001 compared to the no peptide (0 µM) sample. **B** ITC thermogram of CATH-B1 binding ODN-2006. ITC analysis data were calculated as Kd = 6.4 × 10^−8^ M, ΔH = − 65.5 kJ/mol and ΔS = − 73.5 J/mol.

Extracellular microbial DNA is an important signaling molecule in infection and inflammation. Bacterial DNA can be released from phagolysosomes after phagocytosis and bacterial degradation by macrophages, leading to activation of bystander macrophages [24]. CATH-2 has been shown to increase uptake of extracellular DNA and boost subsequent TLR9 or TLR21 activation [11]. To investigate whether CATH-B1 enhances DNA-induced macrophage activation, as shown before for CATH-2, HD11 cells were incubated with ODN-2006 in the presence or absence of CATH-B1 and CATH-2 as control. HD11 cells were used in these experiments since they are better NO producers than primary macrophages. ODN-2006-induced NO production was determined by the Griess assay. HD11 cells did not produce NO without stimulation nor did cathelicidins alone induce NO, whereas high concentration of ODN-2006 (40 nM) strongly increased the NO production (data not shown). The ODN-2006-induced NO production was clearly enhanced by the presence of CATH-2 and CATH-B1, although a higher concentration of 5 μM CATH-B1 was needed to reach maximal NO production compared to CATH-2 (Figure 8A). This shows that although the overall effect of CATH-B1 on stimulation of macrophages by APEC is inhibitory, the potential to increase stimulation by enhancing uptake of bacterial DNA is also present. For CATH-2, it was shown that the increased response of macrophages towards ODN-2006 was depended on direct binding of CATH-2 to ODN-2006 [11]. Using ITC, it was shown that CATH-B1 indeed also strongly binds ODN-2006 (Figure 8B) with a Kd-value of 64 nM. This binding between CATH-B1 and DNA was enthalpy-driven (ΔH = − 65.5 kJ/mol) with a negative entropy value (ΔS = − 73.5 J/mol).

## Discussion

So far, four cathelicidins have been characterized in chicken, CATH-1, -2, -3, and -B1. In this study, we showed that APEC upregulates gene expression of CATH-B1 in macrophages but not of CATH-1, -2, -3. Recently, it was found by our group that CATH-B1 has strong antiviral activity against influenza A viruses in vitro [14]. In this study, the immunomodulatory effect of CATH-B1 on APEC-, LPS- or ODN-2006-activated chicken macrophages was explored.

The expression of cathelicidins is regulated by many factors including inflammatory and microbial stimuli. In our study, APEC infection enhanced gene expression of CATH-B1 but no or limited upregulation of the other three cathelicidins genes was detected in chicken macrophages. CATH-1, -2, -3, in contrast to CATH-B1, are mainly expressed in heterophils, indicating that CATH-B1 gene expression is regulated separately from CATH-1, -2, -3 and might play a non-redundant role in macrophages. Similarly, butyrate which has known immunostimulatory activity was previously shown to enhance gene expression of CATH-B1 (but not CATH-1, -2, -3) in chicken primary monocytes. Butyrate also enhanced antibacterial activity of primary monocytes against *S. enteritidis* [20]. In contrast to APEC infection, CATH-B1 gene expression was actually downregulated in peripheral blood leukocytes from a *Salmonella typhimurium*-infected chicken [25], but its expression was not affected by *Campylobacter jejuni* infection, suggesting that regulation of CATH-B1 expression is dependent on the infecting bacterial species, and likely also on the cell type studied. However, since gene expression and protein production do not always correlate, it is still needed to determine the localization and amount of CATH-B1 in different tissues and cells in normal and stimulated conditions.

Antimicrobial activity is an important function of cathelicidins. CATH-1, -2, -3 showed good anti-APEC activity, which is consistent with the described broad antimicrobial activity of CATH-1, -2, -3 against a set of both Gram-positive and Gram-negative bacteria [26]. Compared to the other three cathelicidins, the antimicrobial activity of CATH-B1 is less studied. CATH-B1 has only been tested against a limited number of bacterial strains including *E. coli*, *S. aureus*, and *P. aeruginosa* with minimal inhibitory concentration (MIC) values in the range of 0.63–2.5 μM when tested against a low number of bacteria (2 × 10^3^ CFU) [6]. However, in another study the MIC value of CATH-B1 was as high as 12.8 μM against *E. coli* and *S. aureus* using a higher number of bacteria [27], more closely resembling the results of this study. In addition, CATH-B1 had very weak anti-APEC activity in cell culture conditions, suggesting that direct killing of bacteria might not be the main activity of this cathelicidin.

Many studies have tried to correlate structure, charge and hydrophobicity to the antimicrobial activity of peptides. Chicken CATH-1, -2, -3 are largely unstructured in aqueous solution but can form an α-helical amphipathic conformation in a membrane-mimicking environment [5, 28, 29]. Proline residues often induce a kink in the helical structure of cathelicidins and this kink between the two helices is involved in antibacterial activity [28, 30]. The structure of CATH-B1 has been predicted, but its conformation has not been determined. However, there are some clear differences between CATH-1, -2, -3 and CATH-B1. CATH-B1 is longer and contains a lower number of positively charged residues (Additional file 3). These differences could at least partially explain the observed difference in antimicrobial activity of CATH-B1, but future structure–activity studies should be performed to determine which characteristics of CATH-B1 play a role in its antimicrobial activity.

In addition to direct microbial killing, cathelicidins can exert immunomodulatory effects on host cells. In previous studies, it has been shown that CATH-2 can strongly reduce activation of macrophages by neutralization of bacteria or bacterial products. In fact, it was hypothesized that CATH-2 has a dual role in first killing a pathogen and subsequently reducing an unwanted inflammatory reaction towards the dead bacterium (or its products) [12]. Our results showed that CATH-B1 inhibited both viable- and heat-killed APEC-induced inflammatory responses in macrophages, although CATH-B1 did not actually kill bacteria. Subsequent studies on LPS binding and neutralization of LPS-induced immune responses suggest that CATH-B1 exerted similar anti-inflammatory properties to CATH-2, and several other host defense peptides [31, 32]. Unlike the immunomodulatory functions of the only human cathelicidin LL-37 that are described to be mediated by several receptors [33–35], it is still unknown whether chicken cathelicidins regulate immune response via interaction with specific cell receptors, or only act on bacterial ligands like LPS and DNA. Interestingly, when primary macrophages were preincubated with CATH-B1, upregulation of IL-10 expression was observed in response to APEC infection, suggesting that CATH-B1 might modulate inflammation via interaction with host factors.

Besides anti-inflammatory activity, host defense peptides also exert pro-inflammatory effects on host cells [36]. In our previous study, CATH-2 has been shown to bind to DNA and enhance the DNA-induced TLR9/21 activation of macrophages [11]. Similar to CATH-2, our results in the current study showed that CATH-B1 also enhances DNA-induced NO production in macrophages, likely using a similar mechanism in which the cathelicidin binds to DNA and is taken up as a complex. ITC showed indeed that CATH-B1 strongly binds DNA, with similar entropy-driven binding characteristics as CATH-2. This enhanced response was also induced by other host defense peptides, such as human/porcine cathelicidins and defensins [37–39]. The complexity of the combined cathelicidin pro- and anti-inflammatory activity makes it difficult to predict which activity will play a major role in a given situation in vivo, but it also indicates that it enables cathelicidins to maintain a balanced immune system in the host upon microbial challenge. In this respect an interesting feature of (some) cathelicidins is their ability of so-called silent killing of bacteria [12], in which bacteria are killed by cathelicidins but the unwanted subsequent immune activation by bacterial products is decreased at the same time. Unraveling the combination of anti-inflammatory and pro-inflammatory properties of cathelicidins will provide insight for development of therapeutic immunomodulators effective against microbial infection [40].

In this study, we found that CATH-B1 has no antibacterial activity in cell culture media. This corresponds with previous studies that many cathelicidins lose their antimicrobial activity in the presence of serum or physiological salt concentrations [41, 42]. This means that in vivo other antimicrobial mechanisms are needed to kill bacteria. One such way could be that cathelicidins use their immunomodulatory properties to regulate the immune system. On the other hand, it has been shown that cathelicidins can have synergistic effects with other host-derived antimicrobial agents against invading pathogens, such as lysozyme and lactoferrin [43]. Therefore, participating in bacterial killing in in vivo conditions might still be an important feature of cathelicidins.

In conclusion, these studies show the overall anti-inflammatory effect of CATH-B1 on APEC-infected or LPS-stimulated macrophages. This functional exploration of CATH-B1 provides a useful first set of information that justifies further investigations into the role of this less studied chicken cathelicidin in vivo.

## Supplementary information

**Additional file 1**. **Antibacterial activity of chicken cathelicidins against APEC in cell culture medium**. Bacteria were incubated with different concentrations of cathelicidins in DMEM or RPMI1640 + glutamax containing FCS for 3 h, serially diluted and spread plated on agar media to quantify viable bacteria. **A** Antibacterial activity of cathelicidins in DMEM + glutamax medium containing FCS. **B** Antibacterial activity of 5 μM CATH-B1 in RPMI1640 + glutamax medium containing 10% FCS. Data are represented as mean  ±  SEM of three independent experiments of triplicate samples per experiment.

**Additional file 2**. **The effect of CATH-B1 on LPS-induced IL-6 protein production in mouse macrophages**. RAW cells were incubated with LPS (100 ng/mL) in the presence or absence of 5 μM CATH-2 and CATH-B1. Concentrations of IL-6 in the cell supernatant were determined by ELISA. Data are represented as mean  ±  SEM of three independent experiments of triplicate samples per experiment. *P ≤ 0.05.

**Additional file 3. Characteristics of chicken cathelicidins.**

## Data Availability

All data sets generated for this study are included in the article.

## Abbreviations

APEC: avian pathogenic *Escherichia coli*
CATH: cathelicidin
HDP: host defence peptide
IFN: interferon
IL: interleukin
ITC: isothermal titration calorimetry
LPS: lipopolysaccharide
MBC: minimal bactericidal concentration
NO: nitric oxide
PBMC: peripheral blood mononuclear cells
TLR: toll-like receptor
TSA: tryptic Soy Agar
