# Supplementary figures and images for: The immunomodulatory effect of cathelicidin-B1 on chicken macrophages
Source: Vet Res. 2020 Sep 24;51:122. doi: 10.1186/s13567-020-00849-y (PMC7517697; doi:10.1186/s13567-020-00849-y)

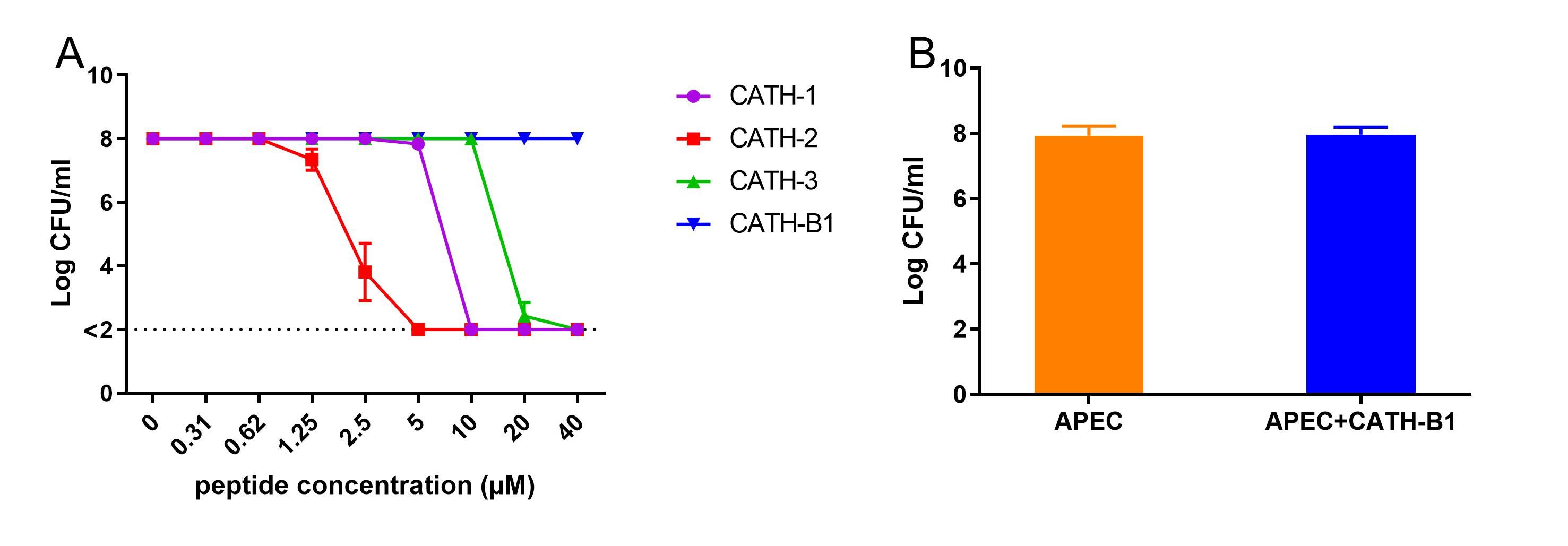

Supplement: Supplementary file 1 — Additional file 1. Antibacterial activity of chicken cathelicidins against APEC in cell culture medium. Bacteria were incubated with different concentrations of cathelicidins in DMEM or RPMI1640 + glutamax containing FCS for 3 h, serially diluted and spread plated on agar media to quantify viable bacteria. A Antibacterial activity of cathelicidins in DMEM + glutamax medium containing FCS. B Antibacterial activity of 5 μM CATH-B1 in RPMI1640 + glutamax medium containing 10% FCS. Data are represented as mean ± SEM of three independent experiments of triplicate samples per experiment. [file 13567_2020_849_MOESM1_ESM.jpg]

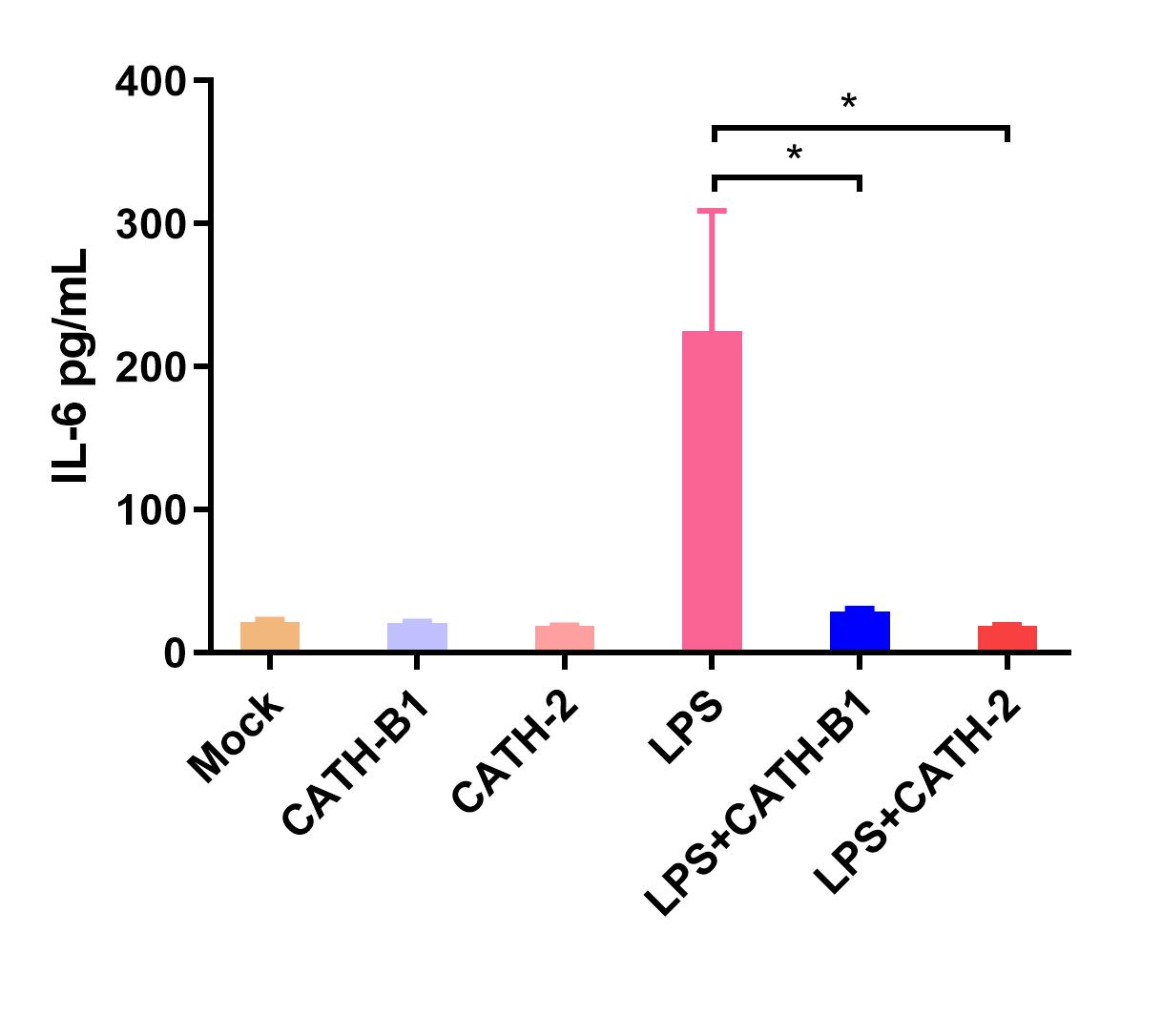

Supplement: Supplementary file 2 — Additional file 2. The effect of CATH-B1 on LPS-induced IL-6 protein production in mouse macrophages. RAW cells were incubated with LPS (100 ng/mL) in the presence or absence of 5 μM CATH-2 and CATH-B1. Concentrations of IL-6 in the cell supernatant were determined by ELISA. Data are represented as mean ± SEM of three independent experiments of triplicate samples per experiment. *P ≤ 0.05. [file 13567_2020_849_MOESM2_ESM.jpg]
