# Supplementary material for: The immunomodulatory effect of cathelicidin-B1 on chicken macrophages
Source: Vet Res. 2020 Sep 24;51:122. doi: 10.1186/s13567-020-00849-y (PMC7517697; doi:10.1186/s13567-020-00849-y)
Supplement: Supplementary file 3 — Additional file 3. Characteristics of chicken cathelicidins. [file 13567_2020_849_MOESM3_ESM.docx]

**Additional Table S1: Characteristics of chicken cathelicidins**

| **Peptide** | **Amino acid sequence** | **length** | **charge** |
| --- | --- | --- | --- |
| **CATH-1** | RVKRVWPLVIRTVIAGYNLYRAIKKK | 26 | +8 |
| **CATH-2** | RFGRFLRKIRRFRPKVTITIQGSARF | 26 | +9 |
| **CATH-3** | RVKRFWPLVPVAINTVAAGINLYKAIRRK | 29 | +7 |
| **CATH-B1** | PIRNWWIRIWEWLNGIRKRLRQRSPFYVRGHLNVTSTPQP | 40 | +7 |
